# Management of in-Amphora “Trebbiano Toscano” Wine Production: Selection of Indigenous *Saccharomyces cerevisiae* Strains and Influence on the Phenolic and Sensory Profile

**DOI:** 10.3390/foods12122372

**Published:** 2023-06-14

**Authors:** Simona Guerrini, Damiano Barbato, Silvia Mangani, Donatella Ganucci, Giacomo Buscioni, Viola Galli, Andrea Triossi, Lisa Granchi

**Affiliations:** 1 FoodMicroTeam s.r.l., Academic Spin-Off of the University of Florence, via Santo Spirito, 14-50125 Florence, Italy; simona@foodmicroteam.it (S.G.); damiano@foodmicroteam.it (D.B.); silvia@foodmicroteam.it (S.M.); giacomo@foodmicroteam.it (G.B.); 2 Department of Agriculture, Food, Environment and Forestry (DAGRI), University of Florence, Via San Bonaventura, 13-50145 Florence, Italy; donatella.ganucci@unifi.it (D.G.); lisa.granchi@unifi.it (L.G.); 3 DREAM Via Enrico Bindi, 14-51100 Pistoia, Italy; triossi@dream-italia.net

**Keywords:** amphorae, indigenous yeast strains, *Saccharomyces cerevisiae*, alcoholic fermentation, wine, polyphenols, Trebbiano Toscano

## Abstract

The use of earthenware amphorae in winemaking can give wines unique attributes enhancing their typicity. Therefore, in this study, spontaneous and inoculated in-amphora fermentations of Trebbiano Toscano grape must were monitored to assess the *Saccharomyces cerevisiae* strains occurring in each fermentation as well as the chemical characteristics of the wines. Strain typing via Interdelta analyses pointed out that the commercial starters did not dominate, showing 24% and 13% implantation percentages, and that 20 indigenous strains were present at different percentages, ranging from 2 to 20%, in inoculated and spontaneous fermentations. The assessment of the technical characteristics of the indigenous strains via fermentations at lab and pilot scale (20 L amphorae) and the sensory analysis of the experimental wines allowed for the selection of two indigenous strains to be used as starter cultures in comparison to a commercial strain in 300-L-amphorae vinifications in the cellar. The observed fermentative performances and sensory analysis of the experimental wines highlighted that one indigenous S. cerevisiae strain dominated the process and conferred distinctive sensory characteristics to the Trebbiano Toscano wine, demonstrating its effectiveness in managing the in-amphora fermentations. In addition, the results demonstrated the ability of amphorae to protect the polyphenolic compounds from oxidation during wine ageing. Indeed, the concentration of both hydroxycinnamic acids and flavonols decreased, with an average reduction of 30% and 14%, respectively, while hydroxybenzoic acids remained unchanged.

## 1. Introduction

Traditional winemaking in amphorae is one of the oldest known methods of wine production [1]. Recently, earthenware or terracotta vessels have received newly gained interest as containers for wine fermentation and ageing because they can give wines unique attributes that enhance their typicity [2]. In addition, earthenware vessels have a relatively low capacity and allow winemakers to produce wines by using small batches of high-quality grapes as raw material, opening a range of economic opportunities for small wine producers to diversify their products [1,3,4]. As a consequence of this new commercial interest, some cultivars have been tested for the production of wines in amphorae, both white grapes such as Sauvignon, Chardonnay, Riesling, Grecanico, Falanghina, and red grapes such as Grenache, Gamay, Aglianico, Nero d’Avola, Frappato, Teroldego [5]. However, most of these studies refer to the physicochemical and sensory characteristics of wines aged in amphorae [5,6,7,8], while just a few are concerned with their microbial ecology [1,9].

Studies on the physicochemical aspects of the wines produced in amphora highlight the differences with the wines aged in vessels of different materials, steel in particular. Indeed, while stainless steel is an inert material and prevents gaseous exchanges, earthenware amphorae are porous [5]. The porosity of earthenware allows oxygen to permeate, resulting in a slow rate of wine oxygenation similar to what occurred in the wooden barrels [1]. However, this statement cannot be generalized as it is related to the production conditions of the amphorae, as reported by Seo et al. [10]. Moreover, very little data are available in the scientific literature on oxygen transmission rate through wood barrels and are almost absent in earthenware vessels [5]. In any case, differences between wine aged in steel, wood, or earthenware seem to be mainly related to the polyphenolic and aromatic components of wine [11]. Different concentrations of oxygen during winemaking can also influence the rate of alcoholic fermentation and the concentration of different compounds produced by yeasts, mainly esters, higher alcohols, medium-chain fatty acids, branched acids, aldehydes, and ketones [12,13,14]. Hence, the use of amphorae could have a significant impact on the metabolism of yeasts and, in particular, of *Saccharomyces cerevisiae*, the yeast mainly responsible for alcoholic fermentation in wine. To the best of our knowledge, there is little available information in the scientific literature that addresses the impact of amphorae on the dynamics of alcoholic fermentation [1]; furthermore, no studies have been conducted to investigate the effectiveness of commercial yeast starters or *S. cerevisiae* biodiversity of spontaneous wine fermentations carried out in these vessels. In general, during the spontaneous fermentation of wine, the development of different indigenous *S. cerevisiae* strains has been widely and demonstrated, and this biodiversity plays an active role in the definition of the sensorial characteristics of the final product [9,15,16,17]. Moreover, indigenous *S. cerevisiae* strains might be better adapted than commercial starters to peculiar fermentative conditions [18,19,20,21]. This could be especially true in wine fermentations carried out in amphorae, as commercial starters have usually been selected for fermentations carried out in steel or concrete tanks. The adequate selection of indigenous *S. cerevisiae* strains might also contribute to the production of wines characterized by a more distinctive flavour and aroma [22], matching the contribution conferred by the use of the amphora to the final product. This is particularly interesting for cultivars, such as Toscano Trebbiano, which is among the most widespread white cultivar in Italy due to its high adaptability to the different types of soil and climatic conditions [23]. Although the resulting wines are generally pleasant and correct, they are often characterized by a neutral profile [24]. The use of amphorae, in association with appropriately selected autochthonous strains, could contribute to the production of wines with a more marked, and therefore more recognizable, sensorial profile. To optimize the wine production process in earthenware amphorae, the first step is to know the effects of this kind of vessel on the wine fermentation and ageing processes from a microbiological and chemical point of view. Therefore, the aim of this study was twofold: (1) to assess the possibility of selecting indigenous *S. cerevisiae* strains for the management of alcoholic fermentation in amphorae by monitoring some spontaneous and inoculated fermentations; (2) to investigate the impact of earthenware amphorae on the phenolic profile of the wine obtained from white grapes of Trebbiano Toscano cultivar.

## 2. Materials and Methods

The experimental design of the research is reported in Figure S1.

### 2.1. Cellar Fermentations to Isolate Indigenous S. cerevisiae Yeasts

Six alcoholic fermentations were carried out in 300-L terracotta amphorae (made in Impruneta, Tuscany, Italy) that were not previously used. Amphorae 1 and 2 were inoculated at 2 × 10^6^ cell/mL with the commercial starter Anchor VIN13, and amphorae 3 and 4 at the same cell concentrations with the commercial starter Aroma White Enartis, while amphorae 5 and 6 were left to ferment spontaneously. These amphorae were oval with a shape that favours the usual internal movements of the wine and were characterized by a uniform thickness of 2.5 cm in all parts in contact with the wine and had a double internal layer of terracotta. The amphorae’s closure consisted of a terracotta system, which guarantees the container’s water tightness, excluding the use of resins and steel. The amphorae were located in a specific area of a Tuscany winery. Grapes of the Trebbiano Toscano variety were collected from the 2021 harvest, and the obtained grape juice was used for the experimental cellar fermentations. The grape juice composition was as follows: glucose, 91 g/L; fructose, 97 g/L; malic acid, 2.98 g/L; total nitrogen, 127 mg/L; total SO_2_, 25 mg/L; pH 3.31. Grape must was characterized by non-*Saccharomyces* yeasts at concentrations lower than 100 CFU/mL, lactic acid bacteria at 1.0 × 10^3^ CFU/mL, nitrogen base 6.7 g/L; *p*-coumaric acid, 100 mg/L; cycloheximide, 10 mg/L; ethanol, 6% *v/v*; bromocresol green, 22 mg/L; agar, 20 g/L). The fermentations were monitored through the use of microbiological and chemical analysis, as reported below. Dissolved oxygen and temperature were monitored (detection intervals every 5 min) in each amphora with Oxilevel Dos 225 sensors commercialized by Parsec Srl (Florence, Italy).

### 2.2. Microbiological Analysis

Yeasts were isolated in four different moments of the various alcoholic fermentations from the plates of WL Nutrient Agar medium. After the purification of the colonies through successive streaking, the yeasts were grown on YPD medium 1% (*w/v*) yeast and acetic acid bacteria at 1.3 × 10^3^ CFU/mL. During the alcoholic fermentations, the yeasts were quantified on WL Nutrient Agar medium, and the plates were incubated for 48 h at 30 °C under aerobic conditions. Lactic acid bacteria were quantified on MRS Agar medium and incubated for five days at 30 °C under anaerobic conditions, and acetic acid bacteria were quantified on Lafon-Lafourcade medium at 30 °C under aerobic conditions, *Brettanomyces bruxellensis* was quantified on DBDM medium (*Dekkera*/*Brettanomyces* Differential Medium).

### 2.3. Identification and Typing of Saccharomyces cerevisiae Strains

Almost 500 yeast isolates were identified as belonging to the *Saccharomyces cerevisiae* species via the amplification of the 5.8S rRNA gene and of the two ribosomal internal transcribed spacers, as described by Granchi et al. [25]. All of these isolates were also characterized at the strain level via inter-δ PCR typing, as reported by Legras and Karst [26], with a δ12/δ21 primer pair [26]. To distinguish the indigenous *S. cerevisiae* strains, the following commercial starters commonly used in the cellar were also analysed: EnartisFerm, Aroma White, EnartisFerm TT EnartisFerm D20 (Enartis, Trecate, Novara, Italy); VIN 13, Alchemy I (Anchor Oenology Cape Town, South Africa); Fermivin PDM (Corimpex service s.r.l. Romans d’Isonzo, Gorizia, Italy); Zymaflore X16, Zymaflore F83; Zymaflore F15, Zymaflore FX 10, Zymaflore RB4, Actiflore D-ONE (Laffort, Bordeaux, France); Viw smart, Viw Fresh, Viw fruity, Viw clever, (Enologica Vason S.p.A. S.Pietro in Cariano, Verona, Italy). The genomic patterns were submitted for pairwise comparison using the Dice coefficient and cluster analysis with the unweighted pair group method. *S. cerevisiae* diversity in each fermentation was quantified by using the two indices “H” and “e” [27].

### 2.4. Technological Characterization of S. cerevisiae Strains

The technical characteristics, including killer character, capability to produce hydrogen sulphide, β-glucosidase activity, and protease activity, were preliminarily investigated to select indigenous *S. cerevisiae* strains with proper oenological features for wine production.

#### 2.4.1. Killer Activity Tests

Killer activity tests were performed according to Philliskirk and Young [28]. The reference sensitive strain *S. cerevisiae* (NCYC 1006; National Collection of Yeast Cultures, Norwich, UK) and the killer reference strain *S. cerevisiae* NCYC 738 (National Collection of Yeast Cultures, Norwich, UK) were used.

#### 2.4.2. Hydrogen Sulfide Production

The capability to produce hydrogen sulfide was estimated, as reported by Aponte et al. [29], on BIGGY agar plates. After incubation at 26 °C for 72 h, H_2_S production was estimated via colony blackening after 3 days of incubation at 28 °C, using a five-level scale from 0 (white) to 5 (black).

#### 2.4.3. β-glucosidase Activity

β-glucosidase activity tests were performed according to Hernández et al. [30] on an Agar esculin medium, pH 5. A single colony was streaked onto the plate surface and was incubated at 30 °C for 48–72 h. Strains possessing β-glucosidase activity produced a brown halo around the streak; a five-level scale was used, from 0 (no halo) to 5 (dark brown halo).

#### 2.4.4. Protease Activity

Protease activity was assayed on Skim milk agar plates (Oxoid ltd, Basingstoke, UK). The plates were inoculated with fresh yeast cultures and incubated at 26 °C for 3 days. Clear halos around the yeast streaks were indicative of proteolytic activity [31].

#### 2.4.5. Fermentative Capacity

The selected strains were tested for their fermentative capacity on the Trebbiano grape must, which was obtained from the 2021 harvest and stored frozen at −20 °C until use. Then, 250-mL Erlenmeyer flasks, sealed with a Müller valve and containing 160 mL of the grape must, were inoculated with the indigenous strains to achieve an initial cell concentration of ca 2 × 10^6^ CFU/mL and incubated at 25 °C. The fermentations were monitored daily, recording the weight loss until the end of the fermentation [22]. The fermentations were carried out in duplicate. The specific growth rate μ (h^−1^) of each assayed strain was calculated by interpolating the data to the Gompertz function. The substrates and products of the main metabolism of yeasts were detected at the end of the fermentations via HPLC, as reported below.

### 2.5. Lab-Scale Fermentations

Based on the results obtained from the preliminary screening, the selected *S. cerevisiae* strains were inoculated at 2 × 10^6^ cell/mL as axenic cultures in 20 L amphorae (made in Impruneta, Tuscany, Italy) containing 15 L of Trebbiano Toscano grape must obtained from the grapes collected on the 2021 harvest. The physicochemical composition of the grape must was as follows: glucose, 90 g/L; fructose, 84 g/L; malic acid,1.10 g/L; total acidity, 5.2 g/L; total nitrogen, 121 mg/L; total SO_2_, 20 mg/L; pH 3.26. The fermentations were conducted in an experimental cellar and monitored via HPLC to determine the main microbial metabolites. At the end of the fermentations, yeasts, lactic acid bacteria, and acetic bacteria were quantified, as reported above. A significant number of colonies grown on the WL medium was randomly picked up from each wine and subjected to inter-δ regions analysis to assess if the inoculated yeast strains were responsible for carrying out the alcoholic fermentation. At the end of the alcoholic and malolactic fermentations, the wines were stabilized to 4 °C and then transferred to glass bottles and placed at 18 °C.

### 2.6. Cellar Fermentations

Grapes of the Trebbiano Toscano variety were collected from the 2022 harvest, and the obtained grape juice was used for experimentation in the cellar. The selected indigenous *S. cerevisiae* strains were inoculated as axenic cultures (Amphora A, initial concentration of 5.0 × 10^6^ CFU/mL or together (Amphora B, initial concentration of 5.0 × 10^6^ CFU/mL) in 300-L amphorae containing Trebbiano Toscano grape must (pH: 3.73). A commercial starter strain was also inoculated under the same conditions as a comparison (Amphora C). The chemical composition of the grape juice was as follows: glucose, 83 g/L; fructose, 98 g/L; malic acid, 1.66 g/L; acetic acid, 0.06 g/L; total nitrogen, 136 mg/L; total SO_2_, 25 mg/L. At the inoculation time, the grape must contained 1.60 × 10^6^ CFU/mL of non-*Saccharomyces* yeasts and concentrations of lactic acid and acetic bacteria below the detection limit. The fermentations were chemically and microbiologically monitored, as reported above. At the end of the alcoholic and spontaneous malolactic fermentations, the wines were bottled and subjected to sensory analysis after three weeks.

### 2.7. Chemical Analysis

Glucose, fructose, ethanol, glycerol, 2,3-butanediol, acetic, lactic, and succinic acid contents in the must and wine were determined via HPLC, according to Guerrini et al. [32], utilizing a Rezex ROA-Organic Acid H+ (8%) column (8-μm particle, 300 × 7.8 mm; Phenomenex, Torrance, CA, USA) and a ProStar 210 chromatograph equipped with a DAD at 210 nm and a Refractive Index Detector, in series (Varian Inc., Palo Alto, CA, USA). The malic acid concentration was determined enzymatically through an automatic multi-parametric analyser (Hyperlab, Steroglass, San Martino, Italy).

### 2.8. Phenolic Compounds Determination

For the determination of hydroxybenzoic and hydroxycinnamic acids, flavonols, flavan-3-ols, stilbenes, and phenolic alcohols, the wines were filtered (0.45 μm) and injected into the HPLC-UV/FLD Jasco series 4000 (Jasco, Japan Spectroscopic co, Hachioji City, Japan) equipped with a pump PU-4180, an autosampler AS-4050, a photodiode array detector MD-4010, and a column oven CO4060 and a reversed-phase column NovaPak C18 (4-μm particle, 300 × 3.9 mm; Waters, Milford, MA, USA), thermostated at 25 °C. The mobile phase was (A) 2% (*v/v*) acetic acid in water and (B) acetonitrile; the gradient profile was 0–40 min, 1–20% B; 40–45 min, 20–50% B; and 45–55 min, 50–95% B, followed by washing with acetonitrile and re-equilibration of the column from 65 to 85 min; the flow rate was 0.9 mL/min from the beginning to 35 min and 1.0 mL/min from this point to the end. Phenolic compounds were detected by scanning from 210 to 600 nm. Hydroxybenzoic acids were quantified at 280 nm using gallic, protocatechuic, vanillic, and syringic acids as standards (Merck Life Science, Milano, Italy); methylgallate and ethylgallate were expressed as gallic acid equivalents. Hydroxycinnamic acids were quantified at 280 nm using caffeic, *trans*-p-coumaric, and ferulic acids as standards (Merck Life Science, Milano, Italy); fertaric acid was expressed as ferulic acid equivalents, trans-caftaric acid as caffeic acid equivalents, *trans*-p-coutaric and *cis*-p-coutaric acids as *trans*-p-coumaric acid equivalents. Stilbenes were quantified at 280 nm using trans-resveratrol and cis resveratrol as standards (Merck Life Science, Milano, Italy); *trans*-piceid and *cis*-piceid were expressed as trans-resveratrol and cis-resveratrol equivalents. Flavan-3-ols were quantified at 280 nm using catechin and epicatechin as standards (Merck Life Science, Milano, Italy); epicatechin-3-O-gallate was expressed as epicatechin equivalents. Flavonols were quantified at 360 nm using quercetin, myricetin, kaempferol, quercetin-3-O-glucoside, quercetin-3-O-glucuronide, quercetin-3-O-galactoside, kaempferol-3-O-glucoside (Merck Life Science, Milano, Italy), myricetin-3-O-glucoside and myricetin-3-O-galactoside (Extrasynthese, Cedex, France) as standards. Phenolic alcohols were quantified at 280 nm using tyrosol and tryptophol as standards (Merck Life Science, Milano, Italy); hydroxytyrosol was expressed as tyrosol equivalents. Volatile phenols (4-vinyl-phenol, 4-vinylguaiacol, 4-ethylphenol and 4-ethylguaiacol) were determined via HPLC-UV/FLD Jasco series 4000 (Jasco, Japan Spectroscopic co, Hachioji city, Japan) equipped with a pump PU-4180, an autosampler AS-4050, a photodiode array detector MD-4010 at 280 nm, a fluorescence detector FP-4025 (λexc 260/λems 305), a column oven CO4060 and a reversed-phase column Kinetex (5-μm particle, 150 × 4.6 mm; Phenomenex, Torrance, CA, USA), and a thermostated at 25 °C. The mobile phase was (A) 0.1 % (*v/v*) phosphoric acid in water and (B) acetonitrile; the gradient profile was 0–25 min, 10–90% B; 25–30 min, 90–10% B, followed by 15 min re-equilibration of the column; the flow rate was 1.0 mL/min.

### 2.9. Biogenic Amines Determination

Biogenic amines (Agmatine, ethanolamine, phenylethylamine, cadaverine, histamine, tyramine, spermine and spermidine) were quantified as damsel-derivatives, as described by Tuberoso et al. [33], using heptylamine as an internal standard. The determination was carried out with an HPLC- UV/FLD Jasco series 4000 (Jasco, Japan Spectroscopic co, Hachioji city, Japan) equipped with a pump PU-4180, an autosampler AS-4050, a photodiode array detector MD-4010, a fluorescence detector FP-4025, and a column oven CO4060 equipped with a 150 mm × 4.6 mm × 5 µm Gemini^®^ C18 column (Phenomenex Inc., Torrance, CA, USA) protected by a C18 SecurityGuard^®^ cartridge. Quantification was performed using calibration curves obtained according to the internal standard method, which correlates the analyte/IS peak area ratio with the concentration. Biogenic amine standards, heptylamine, and dansyl chloride were from Merck (Merck Life Science, Milano, Italy).

### 2.10. Sensory ANALYSIS

The sensory analysis of the wines produced in 300-L and 20-L amphorae were performed strictly according to the methods reported by the Resolution OIV/CONCOURS 332A–2009 [34]. Fifteen panelists were recruited, and the descriptors used were olfactory frankness, olfactory intensity, olfactory quality, gustatory frankness, gustatory intensity, gustatory quality, gustatory persistence, and general impression. The results were expressed on a 5-point scale.

### 2.11. Statistical Analysis

Analytical determinations, performed in duplicate, were elaborated according to nonparametric ANOVA followed by Tukey’s Test. Differences were reported at a significance level of *p* < 0.05 or *p* < 0.01. Principal Component Analysis (PCA) was used to classify the sensorial attributes of the wines. Data of the technological *S. cerevisiae* traits were elaborated through hierarchical cluster analysis (Unweighted Pair Group Method, UPMGA) using the Pearson coefficient. All of the statistical analyses were performed using the Statistica 7.0 software package (Stasoft GmbH, Hamburg, Germany). The genomic patterns were analyzed for pairwise comparison using the Dice coefficient and cluster analysis with the UPGMA method via Gel Compare 4.0 software (Applied Math, Kortrijk, Belgium).

## 3. Results

### 3.1. Alcoholic Fermentation Kinetics in 300 L Amphorae

**Figure 1:**
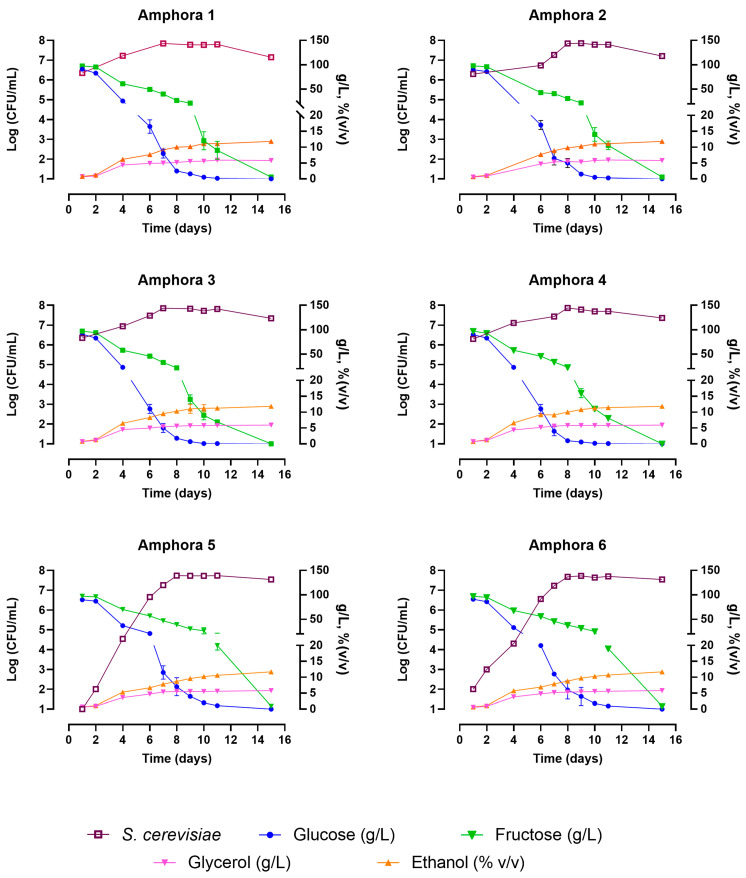
Chemical parameters and *S. cerevisiae* growth kinetics in wine fermentations carried out in 300 L amphorae. Amphorae 1 and 2 were inoculated with the commercial starter Anchor VIN13, amphorae 3 and 4 with the commercial starter Aroma White Enartis, and amphorae 5 and 6 were left to ferment spontaneously.

Grapes of the Trebbiano Toscano variety were collected during the 2021 harvest, crushed and distributed in six amphorae located in a cellar and equipped with probes to monitor dissolved oxygen and temperature. Amphorae 1 and 2 were inoculated with the commercial starter Anchor VIN13, amphorae 3 and 4 with the commercial starter Aroma White Enartis, while amphorae 5 and 6 were left to ferment spontaneously. The chemical parameters of the alcoholic fermentations and the growth kinetics of *S. cerevisiae* populations obtained in each amphora are reported in Figure 1. Populations of non-*Saccharomyces* yeasts and acetic and lactic acid bacteria were present at negligible values (<10^3^ CFU/mL) during the first two days of all the assayed fermentations and then further decreased by reaching values below the detection limit (<10 CFU/mL). In all the fermentations, glucose and fructose were degraded in almost two weeks producing about 12% (*v/v*) of ethanol and acetic acid less than 0.3 g/L. Both spontaneous fermentations (Amphorae 5 and 6) showed regular courses of fermentations with the growth of the *S. cerevisiae* population that reached a concentration of about 6 × 10^7^ CFU/mL in seven days. The regular time course of these spontaneous fermentations suggested the possibility of using the selected indigenous strains belonging to the species *S. cerevisiae* with growth characteristics suitable for producing wines in amphora.

**Figure 2:**
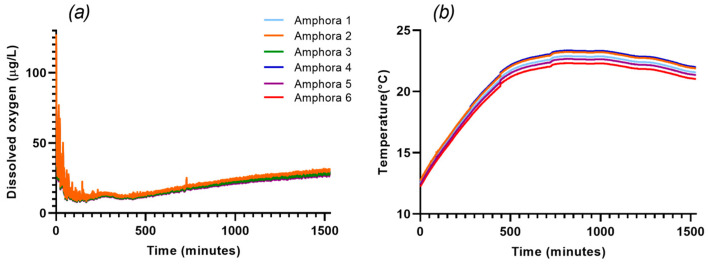
Dissolved oxygen (**a**) and temperature (**b**) of the first 24 h of the six alcoholic fermentations carried out in amphorae.

The monitoring of dissolved oxygen and temperature during the alcoholic fermentation in the first 24 h showed similar trends in all the amphorae (Figure 2). During the first four days of fermentation, and after a period necessary for the must homogenization and the stabilization of the probes, dissolved oxygen ranged between 10 and 15 μg/L. Afterwards, this parameter gradually increased until reaching values between 20 and 25 μg/L at the end of alcoholic fermentation, probably because of the almost daily punching down of the wines. Regarding the temperature trends, all the amphorae showed a rapid increase, around the third day of fermentation, until values between approximately 22 and 24 °C, and in the following days, they remained at values of 22–20 °C. These values of temperature demonstrated the optimal amphorae capacity to maintain the fermenting must at suitable temperatures for the physiology of *S. cerevisiae*.

### 3.2. Chemical and Microbial Characteristics of the Wines during Ageing in 300 L Amphorae

After the end of alcoholic and malolactic fermentations, the wines of each amphora were racked to remove the lees and put back into the clean amphorae to start the ageing. Since the amphorae were filled with the grape must to 80% of capacity, while ageing should be accomplished in full containers, the amphorae were filled as follow: wines obtained with starter Anchor VIN13 were put together in the first amphora (sample coded A1+2); wines obtained with starter Aroma white Enartis in the second amphora (sample coded A3+4) and wines obtained by spontaneous fermentation in the third amphora (sample coded A5+6). Thus, the obtained wines were monitored via physicochemical and microbiological analyses at 4, 6, and 12 months of ageing in amphorae. During this time, the oxygen content in the various amphorae always remained at low levels, precisely below 50 µg/L.

Regarding the evolution of the principal oenological parameters during ageing, wines in the various amphorae showed very similar trends (Table 1): sugars, ethanol, glycerol, and lactic acid concentrations were unaffected, whereas acetic acid increased, even if remaining below the sensory perception threshold (1 g/L). Similarities between the three amphorae were also found as regards the microbiological aspects (Table 1): *S. cerevisiae* and *Oenococcus oeni* populations gradually decreased during ageing until below the detection limit, whereas *Brettanomyces bruxellensis* yeasts were found after 4 months of ageing. To avoid the risk of further growth of this spoilage yeast, sulfites were added (50 mg/L of SO_2_). This operation led over time to the *Brettanomyces* population being below the detection limit in subsequent samplings. Finally, all the vinifications carried out in amphorae showed the complete degradation of malic acid a few days later, the end of alcoholic fermentation, as shown by the lactic acid concentration (Table 1). Since indigenous lactic acid bacteria are usually considered to be mainly responsible for the production of biogenic amines (BAs) in wine, the formation of these compounds was also evaluated. The results reported in Table S1 show a scarce presence of BAs in all the in-amphora assayed wines.

**Figure 3:**
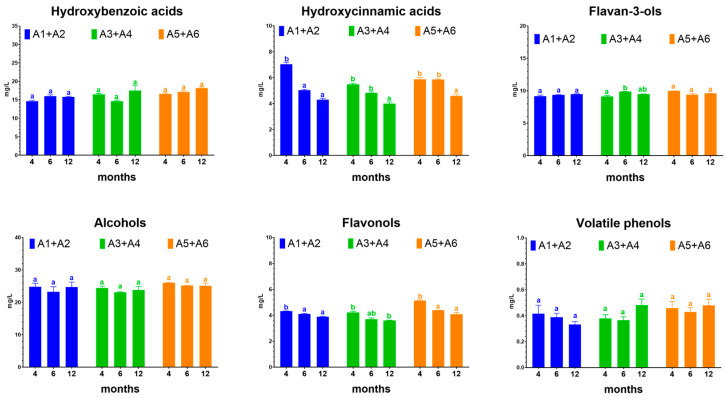
Evolution of the phenolic compounds of the wines during ageing carried out in 300-L amphorae. Different letters indicate significant differences among the same amphora sample collected at different ageing times. (ANOVA, Tukey test at *p* < 0.01).

The phenolic composition of the wines aged in amphorae was monitored for 12 months (Table S2), and Figure 3 shows the evolution of the principal phenolic classes. Stilbenes were not reported because they were detected only at no significant levels (<0.5 mg/L, Table S2). The concentration of both hydroxycinnamic acids and flavonols decreased during the 12 months with an average reduction of 30% and 14%, respectively. On the contrary, the content of hydroxybenzoic acids, flavan-3-ols, and alcohols, which are the phenolic compounds most present in these wines, remained unchanged. No variation was also observed for the phenolic compounds of microbial origin: phenolic alcohols and volatile phenols (Figure 3). Phenolic alcohols (tyrosol, tryptophol, and hydroxytyrosol) are mainly produced by *S. cerevisiae* during alcoholic fermentation from tyrosine and tryptophan, while volatile phenols (vinyl and ethyl phenols) are mainly produced by *Brettanomyces* and are responsible for some sensorial defects in the wine. However, the concentration of volatile phenols was below the sensory threshold.

### 3.3. Biodiversity of Saccharomyces cerevisiae during Alcoholic Fermentation in 300 L Amphorae

The sampling of the yeast microbiota was performed when *S. cerevisiae* populations occurred at concentrations higher than 10^6^ CFU/mL at four different points of the alcoholic fermentation process. The intraspecific characterization via inter-δ regions analysis was carried out on almost 500 *S. cerevisiae* isolates. To recognize indigenous *S. cerevisiae* strains, the inter-δ patterns obtained were compared with the patterns of some commercial yeast starters. In particular, they were compared with the Anchor VIN13 and Aroma White Enartis strains used in this experiment and with the starter strains normally used in the cellar to carry out industrial vinifications. The comparison pointed out 20 different patterns recognized as indigenous *S. cerevisiae* strains, and their isolation frequencies are reported in Table 2. Both commercial starters used in amphora vinifications were not able to completely dominate the fermentation process. Indeed, the strain Anchor VIN13, inoculated in amphorae 1 and 2, showed an isolation frequency of about 27%, while Aroma White Enartis, inoculated in amphorae 3 and 4, showed a lower isolation frequency (about 13%). Two additional commercial yeast strains usually used in the winery were found at low percentages (6–7%) in the inoculated amphorae (Table 2). Moreover, in such amphorae, different indigenous *S. cerevisiae* strains occurred at various percentages. In particular, in amphorae 3 and 4, strain XII was present at a higher percentage than the commercial strain inoculated (20 vs. 13%). The low levels of dominance of the inoculated commercial starters as well as the presence of native *S. cerevisiae* strains with high frequency, suggest the need to select indigenous *S. cerevisiae* strains that are likely better adapted to the specific fermentation conditions and thus more suitable for vinification in amphora. Nevertheless, spontaneous fermentations carried out in amphorae 5 and 6 did not show significant dominance of a limited number of indigenous strains, as their isolation frequencies ranged from about 2 to 13%. Anyway, it is underlined that only in the spontaneous fermentation carried out in the amphora 6, one commercial starter culture, the strain Anchor VIN13, was found but at a low frequency (9%) (Table 2). Finally, to compare the genetic diversity levels of the native *S. cerevisiae* populations isolated from the six vinifications conducted in amphorae, the average values of the Shannon (H) and Evenness (e) indices were calculated, excluding the starter strains (Table 2). The Shannon index (H) measures the diversity within a population, taking into account both the number of diverse genetic profiles and the number of isolates showing the same genetic profile, while Evenness (e) indicates the relative abundance of the diverse strains. The Evenness values range from 0 to 1, with 1 displaying that all strains occur at the same percentage and values close to 0 mean the presence of strains at higher percentages or dominant strains. As expected, the fermentations carried out spontaneously (amphorae 5 and 6) showed greater biodiversity (H) than the others inoculated with commercial starters (amphorae 1, 2, 3, 4). Conversely, all of the amphorae showed high evenness values, close to 1, underlining the absence of indigenous strains with a marked dominance over the others.

**Figure 4:**
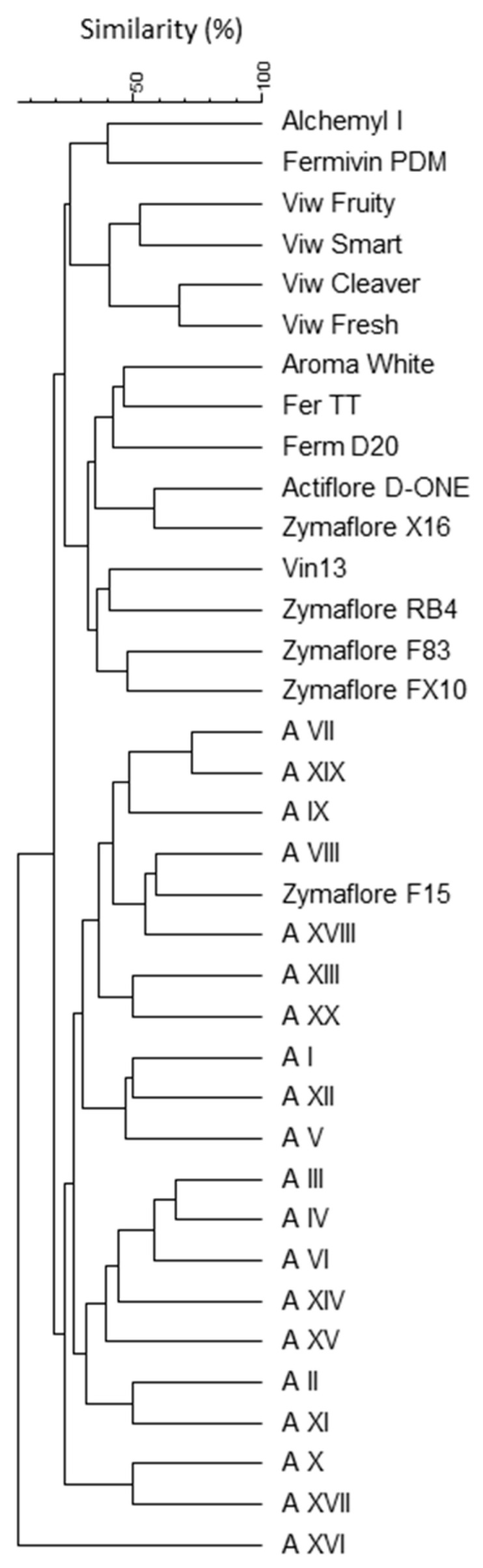
UPGMA dendrogram based on the Dice coefficient of the inter-δ patterns of the indigenous *S. cerevisiae* strains isolated from the fermentations carried out in amphorae and the commercial starters.

The cluster analysis of the different inter-δ patterns highlighted the high genetic distance between 20 indigenous *S. cerevisiae* strains and the commercial starter strains since they grouped into two different clusters at a similarity index of about 20% (Figure 4). Only the commercial starter Zymaflore F15 was grouped into the cluster that included the indigenous strains.

### 3.4. Selection of Indigenous S. cerevisiae Strains

The 20 indigenous *S. cerevisiae* strains were preliminarily screened for the following phenotypic characteristics of oenological interest: killer character, capability to produce hydrogen sulphide, β-glucosidase activity, and protease activity. To summarize these results, a heatmap with the standardized data of the screening was carried out (Figure S2). The heatmap also included the average isolation frequencies found for each strain in spontaneous or inoculated fermentations realized in amphora. These frequencies can be interpreted as a measure of the adaptability of these strains to the amphorae environment.

**Figure 5:**
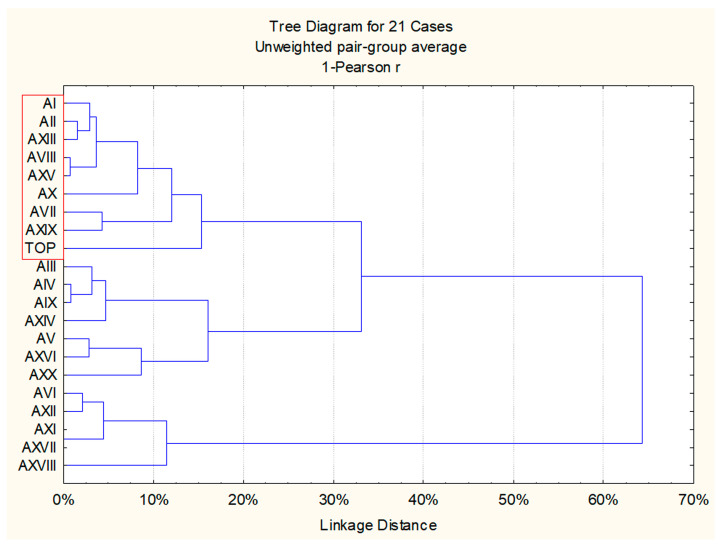
Cluster analysis of the indigenous *S. cerevisiae* strains features: killer character, capability to produce hydrogen sulphide, β-glucosidase activity, protease activity, isolation average frequencies found in spontaneous or inoculated fermentations realized in amphora.

The same data were also elaborated with cluster analysis to select six strains to further test as potential starters (Figure 5). To carry out the selection of indigenous *S. cerevisiae* strains based on their properties, one virtual strain, called “TOP”, was included in the experimental data. The virtual results ascribed to the “TOP” strain were as follows: the presence of killer character, low capacity to produce hydrogen sulphide, presence of β-glucosidase activity, presence of protease activity, and the maximum value of the isolation frequencies among those recorded for indigenous strains. The strains that clustered with the “TOP” strain (indicated by the red square) were those that most closely resembled the ideal starter (Figure 5). Hence, the *S. cerevisiae* strains AI, AII, AVII, and AVIII, which occurred only in spontaneous fermentation, and AXV, and AXIX, as representative of the strains present both in spontaneous and inoculated fermentations, were chosen for further tests in laboratory-scale fermentations.

The six indigenous *S. cerevisiae* strains were tested through 160 mL fermentations on Trebbiano Toscano grape juice to evaluate strain-specific oenological and technological properties. Each strain was singly inoculated at the concentration of 2 × 10^6^ CFU/mL. The results of chemical analyses performed at the end of each alcoholic fermentation (after 10 days from the yeast strain inoculum) are reported in Table 3. After this period, the sugar consumption was completed with low acetic acid production (<0.3 g/L) for all of the tested strains. These strains also demonstrated high fermentative vigour (CO_2_ produced in 48 h higher than 6 g/100 mL) and SO_2_ production lower than 50 mg/L. Other statistical differences between the strains were related to polyalcohol (glycerol and 2,3-butanediol), lactic acid, and succinic acid concentrations. Finally, the Gompertz model was used to estimate the fermentative performance of the strains in terms of specific growth rate (μ-max) (Table 3). The goodness of fit of this model was appropriate for all the assayed strains, with R values being higher than 0.98. The findings pointed out that the μ-max value of the AVIII strain was significantly higher than the other strains. Therefore, the strain AVIII was further characterized through fermentations on a laboratory scale using small amphorae (volume of 20 L). Two additional indigenous strains, AI and AXIX, which showed a suitable fermentation rate, and the commercial starter Anchor VIN13 were also included as a comparison.

### 3.5. Fermentation Performances of Selected Yeast Strains in 20L Amphorae Fermentations

The indigenous strains AI, AVIII, and AXIX and the commercial strain Anchor VIN13 were used as starters in lab-scale fermentations carried out in 20 L amphorae, each containing 18 L of Trebbiano Toscano grapes must. These amphorae were identical to 300 L amphorae but smaller. Each strain was inoculated, as axenic culture, at the concentration of 2 × 10^6^ CFU/mL, and the fermentation kinetics were monitored by quantifying ethanol and sugar concentrations (Figure S3a). The strains AVIII, AXIX, and Anchor VIN13 showed significantly higher fermentation rates than strain AI. In particular, AVIII and Anchor VIN13 completed the alcoholic fermentation in seven days, AXIX in 11 days, and AI in fourteen days (Figure S3b). At the end of the fermentations, molecular analysis of the yeast populations confirmed the dominance of the inoculated *S. cerevisiae* strains.

Table 4 shows the results of chemical and microbiological analyses performed at the end of each alcoholic fermentation. Strain AVIII produced a higher concentration of glycerol than the Anchor VIN13 commercial starter and AXIX strain, while AI and AXIX strains produced higher concentrations of acetic acid. All the vinifications showed the presence of *O. oeni*, but only in those conducted by AI and AXIX strains was malolactic fermentation accomplished during alcoholic fermentation.

When all the vinifications completed malolactic fermentation, the wines were stabilized at 4 °C for fifteen days, bottled, and after three weeks, subjected to sensory analysis. The sensorial characterization of the wines was performed using the experimental approach proposed by the OIV [34]. The wine with the lower means of the total scores (value of acceptance) was the one obtained with the XIX strain. The perceptive map obtained from PCA applied to the mean intensity data of significant attributes is shown in Figure S4. The total variance explained for the sensory attributes based on the first two significant dimensions was 91%, with PC1 and PC2 accounting, respectively, for 65% and 26%. The wine obtained with the AXIX strain was characterized by a negative correlation with all the descriptors and, in particular, with the so-called “general impression”. On the contrary, the wines obtained with Anchor VIN13 and AVIII strains were characterized by a positive correlation with the gustatory descriptors, while only the wine obtained with the AVIII strain was also positively related with the olfactory descriptors. The wine obtained with the AI strain owned intermediate characteristics between AVIII and VIN13 wines. Therefore, AVIII and AI strains were chosen as indigenous starters to induce alcoholic fermentations in cellar vinification realized carried out in 300-L amphorae.

### 3.6. Use of Indigenous S. cerevisiae Strains in Cellar Vinifications Carried out in 300 L Amphorae

**Figure 6:**
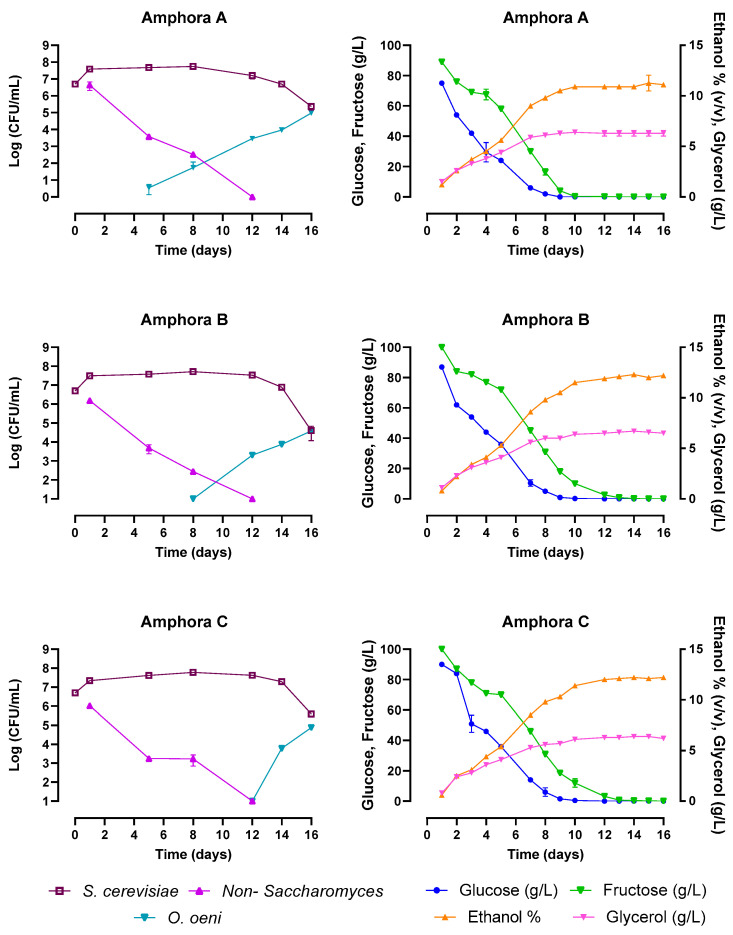
Monitoring of the alcoholic fermentations conducted in real vinification carried out in 300-L amphorae. Amphorae A was inoculated with strain AVIII, Amphora B with strain AVIII and AI, Amphora C with the commercial starter Anchor VIN13.

Grapes of the Trebbiano Toscano variety were collected during the 2022 harvest, crushed, and distributed in the same 300-L amphorae used in the previous harvest. The indigenous strains AI and AVIII and the commercial strain Anchor VIN13 were used as starters. The AVIII strain and the Anchor VIN13 strain were inoculated as axenic culture (Amphora A and C, respectively), while the AI strain was co-inoculated with the AVIII strain (Amphora B). In Figure 6, the chemical and microbiological data collected during the wine fermentations monitoring are reported. In Amphora A, alcoholic fermentation was accomplished after ten days, while in Amphorae B and C, after twelve days. In any case, all of the fermentations followed regular courses and acetic acid production at the end of the fermentation was lower than 0.30 g/L. All of the amphorae fermentations showed the disappearance of non-*Saccharomyces* yeasts within twelve days and the growth of an indigenous population of *O. oeni* before the end of the alcoholic fermentation.

Molecular analyses (PCR of inter-δ regions) were carried out on the yeast isolates collected at the end of each alcoholic fermentation. In Table 5, the isolation frequencies of the yeast strains occurring in the three alcoholic fermentations are reported. The strain Anchor VIN13, which was inoculated as a single culture in Amphora C, showed an isolation frequency of about 25%, confirming the low efficiency of this commercial starter to dominate the alcoholic fermentations carried out in amphorae. Indeed, in Amphora C, the indigenous strain VIII predominated, displaying a percentage of 40%. On the contrary, the AVIII strain, when inoculated singly, showed an isolation percentage of 75%, while when coupled with AI, this percentage dropped to 54% (Table 5). In any case, the total isolation percentage of the two selected strains in the mixed culture (AVIII+AI) was 79%, thus demonstrating the greater “fitness advantage” of properly selected indigenous strains compared to Anchor VIN13 in amphorae vinifications. As regards the presence of other indigenous yeast strains, nine genomic profiles of indigenous strains were found. Only two of these biotypes (AXI and AXIX) were also found in the 2021 harvest.

**Figure 7:**
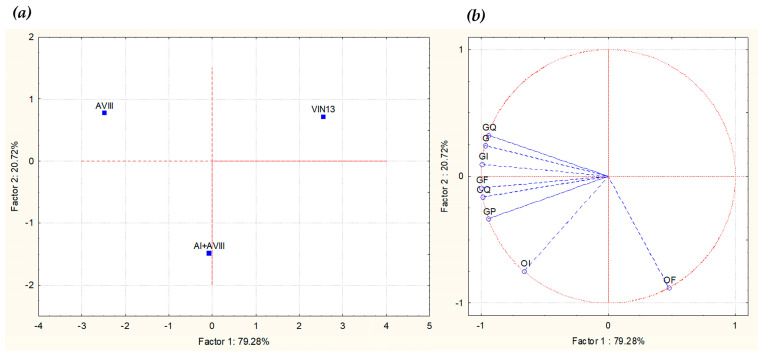
Principal Component Analysis carried out on the sensory analysis of the wines obtained in 300-L amphorae inoculated with the strain AVIII (Amphora A), with the strains AVIII and AI (Amphora B), and with the commercial starter Anchor VIN13 (Amphora C). (**a**): similarity map determined by Principal Component (Factor) 1 and 2; (**b**): projection of the variables on the factor plane. Variables: OF: olfactory frankness; OI: olfactory intensity; OQ: olfactory quality; GF: gustatory frankness; GI: gustatory intensity; GQ: gustatory quality; GP: gustatory persistence; G: general impression.

When all the vinifications also completed malolactic fermentation, the wines were bottled and, after three weeks, subjected to sensory analysis using the method described above. The means of the total scores (value of acceptance) were not significantly different between the three wines, demonstrating that the three resulting wines were free from sensory defects. The perceptive map obtained from PCA applied to the mean intensity data of significant attributes is shown in Figure 7. The first two significant dimensions explained 100% of the total variance, with PC1 and PC2 accounting, respectively, for 79.3% and 20.7%. Only the wines obtained with the selected indigenous *S. cerevisiae* strains (AVIII and AI+AVIII) showed positive correlations with almost all descriptors.

## 4. Discussion

White wines obtained from the grapes of the Trebbiano Toscano cultivar are of widespread consumption in Italy. These wines are generally pleasant and correct but are also characterized by neutral features. The use of amphorae could represent a suitable tool to make these wines more recognizable and appreciated, favouring their positioning on the market. Therefore, this study aimed to investigate the correct management of Trebbiano Toscano grapes vinifications realized in earthenware amphorae by studying the microbiological and physicochemical phenomena that occur during the production of wine. The first aspect taken into consideration was the management of alcoholic fermentation. Therefore, vinifications in earthenware amphorae were realized by letting the must ferment spontaneously or using two different commercial starters selected from those normally used for Trebbiano Toscano grapes in the cellar where the experimentation was carried out. Both spontaneous and inoculated fermentations ended correctly in ten–fifteen days without defects. However, the investigation on *S. cerevisiae* biodiversity showed the incapacity of commercial starter strains, used in the inoculated fermentations, to completely dominate the indigenous *S. cerevisiae* strains as they occurred at frequencies of 27 and 13%. Some authors [35,36,37] reported that the inoculation of a yeast starter has to be considered unsuccessful when the implantation percentage is less than 50–60% and that this percentage can vary according to the commercial starter used as well as the characteristics of grape musts and the vinification technology used in the winery. Aponte et al. [29] observed that different levels of the dominance of commercial yeast starters depend on the fermentation stage in addition to the used commercial starter, and, at the end of fermentation, they detected percentage values similar to those found in this study. Therefore, an adequate choice of commercial starters to use for the vinification management in amphorae should be performed to ensure problem-free fermentation processes. An alternative could be the use of indigenous *S. cerevisiae* strains selected as starters from spontaneous alcoholic fermentations conducted in amphorae. In general, *S. cerevisiae* strains that numerically dominate the spontaneous alcoholic fermentations are more competitive because of their higher capacity to fit the environmental conditions in which they will have to operate [20,21]. Unfortunately, in this study, the fermentations carried out spontaneously showed great biodiversity and the absence of indigenous strains with a marked dominance over the others. However, two indigenous *S. cerevisiae* strains were selected for their enological ability and tested in cellar conditions. The results obtained demonstrate the particular effectiveness of one indigenous *S. cerevisiae* strain (AVIII) for the management of the alcoholic fermentations realized in amphorae. In addition, this selected indigenous strain also conferred distinctive sensory characteristics to the Trebbiano Toscano wine produced in amphorae, thus contributing to characterize the finished products. The advantages of using indigenous strains for the alcoholic fermentation of wines are widely reported in the literature [29,38,39,40,41,42,43,44,45]. However, this study represents the first attempt to use this approach to optimize the production of wines in earthenware amphorae.

The second aspect taken into consideration in this study was the management of wine ageing in earthenware amphorae. In general, during the ageing of wine, the phenolic content decreases as the phenolic compounds undergo hydrolysis, oxidation, and complexation phenomena. The ageing of the wine can be performed in vessels made with different materials such as steel, wood, or earthenware. Stainless steel tanks are inert vessels, while wood and earthenware can physicochemically interact with the wine [6]. When the wine is aged in wood, its sensorial and phenolic profile changes [46]. These changes are due to the wooden barrels’ ability to micro-oxygenate the wine and to release phenolic and aromatic substances while adsorbing other components. However, the ageing of white wines realized in this kind of barrel is not always profitable due to the too-high oxygenation and the risk of an excessive release of substances by the wood that could mask the sensory characteristics of the wine [47]. On the contrary, in-amphorae ageing is an oenological practice suitable to white wines because these vessels can protect polyphenol compounds such as flavonoids, hydroxycinnamoyl tartaric acids, or procyanidins from oxidation and, at the same time, contribute positively to the aromatic characteristics [6]. No information relating to the polyphenolic content during the ageing of wines obtained from grapes of the Trebbiano Toscano cultivar is reported in the literature. In any case, the wines of this study demonstrated a high stability of the polyphenolic content, probably due to the low oxygenation during the ageing. The concentration of dissolved oxygen in the experimental wines of this study was significantly lower than that found in other studies [6], proving the amphorae’s ability to minimize the oxygenation of the wine and consequently protect the polyphenolic component of the wine from oxidation. However, this characteristic cannot be generalized to all amphorae because it depends on their production technique (temperature of fired, typology of internal coating, etc.), as reported by Baiano et al. [5]. Indeed, these authors analyzed the effects of different kinds of amphora on the ageing of wines obtained from Minutolo white grapes. These wines showed more or less significant decreases in flavonoids and hydroxycinnamic acids based on the characteristics of the used amphora [5,6]. In this study, similar trends for hydroxycinnamic acids and flavonoids were found in the amphora-aged wines from Trebbiano Toscano white grape variety.

Despite the low dissolved oxygen content, contaminations by the spoilage yeast *Brettanomyces bruxellensis* were found. This yeast is mainly associated with wines aged in barrels and with stuck or sluggish fermentation [48], even if recent studies indicate the grapes as the possible primary source from which a flow toward the winery environment originates [49]. Therefore, particular attention must be paid to the quality of the grapes used for the fermentation in amphorae, and the detection of *Brettanomyces* during the refinement of the wines is advisable to intervene before the wine is irreparably damaged from this spoilage yeast.

Finally, the wines produced in amphorae during this study showed a particular susceptibility to spontaneous malolactic fermentation. This aspect is perhaps connected to the microaerophilic characteristics of the amphorae. In any case, further studies would be needed to understand the impact of amphorae on the growth kinetics of indigenous malolactic bacteria. This aspect is not only interesting for technological management but also for the hygienic–sanitary effects linked to the possible production of biogenic amines by indigenous malolactic bacteria [50].

## 5. Conclusions

The use of amphorae associated with appropriately selected indigenous strains allowed the production of a Trebbiano Toscano wine with distinctive sensory characteristics and stable polyphenol content. The study also highlights some critical microbiological issues relating to the use of amphorae for the production of wine: the importance of a correct choice of the *S. cerevisiae* starter, the risk of contamination by *Brettanomyces* yeast, and the eventual development of an indigenous population of malolactic bacteria able to produce biogenic amines. These aspects should be taken into consideration for the correct management of wine production in amphorae.

## Figures and Tables

**Table 1:** Chemical and microbiological parameters of the wines during ageing in 300-L amphorae. Different letters indicate significant differences among samples collected at a different ageing time from each amphora (ANOVA, Tukey test at *p* < 0.01).

	A1+2 (Anchor VIN13)			A3+4 (Aroma White Enartis)			A5+6 (Spontaneous)		
Months	4	6	12	4	6	12	4	6	12
Glucose (g/L)	<0.1	<0.1	<0.1	<0.1	<0.1	<0.1	<0.1	<0.1	<0.1
Fructose (g/L)	0.2 ± 0.0 a	0.2 ± 0.0 a	0.2 ± 0.0 a	0.1 ± 0.0 a	0.1 ± 0.0 a	0.1 ± 0.0 a	0.5 b	0.5 b	0.2 a
Ethanol (%, *v/v*)	11.8 ± 0.3 a	11.8 ± 0.1 a	11.7 ± 0.1 a	11.8 ± 0.0 a	11.8 ± 0.0 a	11.7 ± 0.1 a	11.8 ± 0.0 a	11.8 ± 0.1 a	11.9 ± 0.0 a
Glycerol (g/L)	5.8 ± 0.3 a	5.8 ± 0.0 a	5.8 ± 0.1 a	5.7 ± 0.3 a	5.7 ± 0.1 a	5.8 ± 0.0 a	5.8 ± 0.1 a	5.8 ± 0.0 a	5.8 ± 0.1 a
Acetic acid (g/L)	0.23 ± 0.00 a	0.23 ± 0.00 a	0.30 ± 0.01 b	0.21 ± 0.00 a	0.21 ± 0.01 a	0.25 ± 0.01 a	0.18 ± 0.01 a	0.18 ± 0.00 a	0.20 ± 0.01 a
Lactic acid (g/L)	1.87 ± 0.03 a	1.87 ± 0.04 a	1.87 ± 0.07 a	1.83 ± 0.04 a	1.83 ± 0.06 a	1.83 ± 0.00 a	1.95 ± 0.00 a	1.95 ± 0.04 a	1.95 ± 0.04 a
*S. cerevisiae* (CFU/mL)	5.60 × 10^3^ a	6.40 × 10^2^ b	<10	2.40 × 10^2^ a	30 b	<10	3.23 × 10^4^ a	4.9 × 10^2^ b	<10
*O. oeni* (CFU/mL)	7.60 × 10^3^ a	1.66 × 10^2^ b	<10	3.00 × 10^3^ a	1.52 × 10^2^ b	<10	6.56 × 10^4^ a	1.18 × 10^4^ b	<10
*B. bruxellensis* (CFU/mL)	1.60 × 10^3^	<10	<10	1.70 × 10^2^ a	6.10 × 10^2^ b	<10	3.00 × 10^3^	<10	<10

**Table 2:** Isolation frequencies expressed as percentages of the indigenous and starter *S. cerevisiae* strains in the six fermentations carried out in amphorae. Diversity indices were calculated according to Shannon and Weaver [27] for the *S. cerevisiae* population obtained from the different amphorae.

%	Amphora 1	Amphora 2	Amphora 3	Amphora 4	Amphora 5	Amphora 6
*Indigenous* *S. cerevisiae strains*						
AI	-	-	5.6	7.6	6.7	2.2
AII	-	-	6.4	6.8	6.7	4.4
AIII	-	-	-	-	4.4	2.2
AIV	-	-	-	-	4.4	2.2
AV	-	-	-	-	6.7	2.2
AVI	7.5	5.7	14.0	13.0	2.2	6.7
AVII	-	-	7.1	6.1	13.3	11.1
AVIII	-	-	6.7	6.5	4.4	8.9
AIX	-	-	-	-	4.4	-
AX	7.6	5.6	-	-	4.4	4.4
AXI	12.5	14.5	-	-	4.4	4.4
AXII	-	-	20.1	19.9	2.2	4.4
AXIII	7.7	5.5	-	-	2.2	6.7
AXIV	-	-	-	-	2.2	6.7
AXV	7.1	6.1	-	-	4.4	6.7
AXVI	-	-	-	-	6.7	2.2
AXVII	-	-	12.5	14.5	4.4	4.4
AXVIII	13.0	14.0	-	-	2.2	-
AXIX	5.6	7.6	-	-	11.1	8.9
AXX	-	-	-	-	2.2	2.2
*Commercial strains*						
Anchor VIN13	25.6	27.6	6.5	6.9	-	8.9
Aroma White Enartis	5.7	7.7	14.3	12.3	-	-
Zymaflore X16	7.7	5.7	6.8	6.4	-	-
Enartis Ferm TT	-	-	5.6	7.6	-	-
*Biodiversity indeces*						
H	1.87	1.91	1.82	1.86	2.85	2.83
e	0.85	0.87	0.76	0.78	0.75	0.76

**Table 3:** Chemical analyses performed at the end of each alcoholic fermentation on Trebbiano Toscano grape must and fermentative performance of the strains in terms of specific fermentation rate (μ-max) estimated with the Gompertz model. Different letters in the same row indicate statistically significant differences (ANOVA and Tukey’s Test, *p* < 0.05).

Strain	AI		AII		AVII		AVIII		AXV		AXIX	
	Mean	SD	Mean	SD	Mean	SD	Mean	SD	Mean	SD	Mean	SD
Glucose (g/L)	<0.1	-	<0.1	-	<0.1	-	<0.1	-	<0.1	-	<0.1	-
Fructose (g/L)	<0.1	-	<0.1	-	<0.1	-	<0.1	-	<0.1	-	<0.1	-
Ethanol (%, *v/v*)	11.1	0.1	11.2	0.0	11.2	0.0	11.3	0.0	11.2	0.1	11.2	0.1
Glycerol (g/L)	5.8 a	0.1	6.0 a	0.1	5.8 a	0.1	6.1 a	0.0	5.4 b	0.1	5.5 b	0.1
meso 2,3-butanediol (g/L)	0.07 ab	0.01	0.09 a	0.01	0.07 ab	0.01	0.02 b	0.01	0.03 ab	0.03	0.01 b	0.00
raceme 2,3-butanediol (g/L)	0.18 ab	0.01	0.15 ab	0.03	0.13 a	0.03	0.14 a	0.02	0.15 ab	0.01	0.23 b	0.03
Succinic acid (g/L)	1.09 a	0.01	0.86 ab	0.03	0.87 ab	0.01	0.90 ab	0.04	0.75 b	0.11	0.90 ab	0.16
Acetic acid (g/L)	0.21	0.04	0.26	0.01	0.24	0.01	0.20	0.02	0.26	0.01	0.18	0.01
Lactic acid (g/L)	0.20 a	0.01	0.15 ab	0.01	0.17 ab	0.01	0.13 b	0.03	0.16 ab	0.01	0.20 a	0.01
Fermentation rate (μmax, h^−1^)	0.100 a	0.002	0.090 a	0.0004	0.093 a	0.002	0.114 b	0.006	0.097 a	0.008	0.100 a	0.004

**Table 4:** Chemical and microbiological analyses performed at the end of the alcoholic fermentation carried out in 20-L amphorae, and fermentative performance of the indigenous *S. cerevisiae* strains (AI, AVIII,) and the commercial strain Anchor VIN13 in terms of specific fermentation rate (μ-max) estimated with the Gompertz model. Different letters in the same row indicate statistically significant differences (ANOVA and Tukey’s Test, *p* < 0.05).

	AI		AVIII		AXIX		VIN13	
	Mean	SD	Mean	SD	Mean	SD	Mean	SD
Glucose (g/L)	1.10 a	-	0.10 b	0.07	<0.05	-	0.10 b	-
Fructose (g/L)	0.8 ac	0.1	0.3 ab	0.1	0.1 b	0.1	0.9 c	0.1
Ethanol (% *v/v*)	11.3	0.4	10.8	0.4	11.0	0.1	11.4	0.0
Glycerol (g/L)	7.7 ab	0.1	8.1 a	0.1	7.4 b	0.1	7.3 b	0.1
Lactic acid (g/L)	1.23 a	0.14	0.19 b	0.01	1.50 a	0.15	0.12 b	0.03
Acetic acid (g/L)	0.31 a	0.01	0.12 b	0.02	0.30 a	0.03	0.15 b	0.03
Malic acid	<0.05	-	1.94	0.05	<0.05	-	1.97	0.08
Fermentation rate (h^−1^)	0.056 a	0.004	0.190 b	0.008	0.167 b	0.010	0.174 b	0.008
*S. cerevisiae* (CFU/mL)	5.43 × 10^6^ a	0.41 × 10^6^	6.80 × 10^7^ b	0.65 × 10^7^	8.40 × 10^4^ c	0.48 × 10^4^	1.50 × 10^6^ d	0.32 × 10^6^
*O. oeni* (CFU/mL)	4.05 × 10^7^ a	0.09 × 10^7^	9.10 × 10^3^ b	0.15 × 10^3^	8.00 × 10^7^ c	0.12 × 10^7^	8.80 × 10^4^ d	0.60 × 10^4^

**Table 5:** Isolation frequencies expressed as percentages of the indigenous *S. cerevisiae* strains (AVIII, AI) and the Anchor VIN13 strain in the fermentations carried out in Amphorae A, B, and C.

%	Amphora A (AVIII)	Amphora B (AI+AVIII)	Amphora C (VIN13)
AI	-	25	7
AVIII	75	54	40
VIN13	-	-	25
AXI	7	-	-
AXIX	-	-	7
AXXII	11	11	-
AXXIII	-	7	-
AXXIV	-	-	3.5
AXXV	7	-	7
AXXVII		3	-
AXXIX	-	-	3.5
AXXXI	-	-	7

## Data Availability

Data are contained within the article or supplementary material.

## Supplementary Materials

The following supporting information can be downloaded at: https://www.mdpi.com/article/10.3390/foods12122372/s1, Figure S1: Workflow of the research; Figure S2: Heatmap based on indigenous *S. cerevisiae* strains features: killer character (A), capability to produce hydrogen sulphide (B), β-glucosidase activity (C), protease activity (D), average isolation frequencies found in spontaneous (E) or inoculated fermentations (F) realized in amphora. The values of each activity were standardized to range from 0 to 100. Colours correspond to standardized values from low (white) to high (black); Figure S3: Fermentation performances in 20-L amphorae of the selected indigenous *S. cerevisiae* strains (AI, AVIII, AXIX) and the commercial strain Anchor VIN13 monitored by quantifying the production of ethanol and the degradation of total sugars during the time; Figure S4: Principal Component Analysis carried out on the sensory analysis of the wines obtained with different *S. cerevisiae* strains (AI, AVIII, AXIX, VIN13) in 20 L amphorae. (a): similarity map determined by Principal Component (Factor) 1 and 2; (b): projection of the variables on the factor plane. Variables: OF: olfactory frankness; OI: olfactory intensity; OQ: olfactory quality; GF: gustatory frankness; GI: gustatory intensity; GQ: gustatory quality; GP: gustatory persistence; G: general impression. Table S1: Biogenic amine concentrations occurring in the wines during ageing in 300-L amphorae. Different letters indicate significant differences among samples collected at a different ageing time from each amphora (ANOVA, Tukey test at *p* < 0.01); Table S2: Phenolic composition of the wines aged in amphorae was monitored for 12 months.
