# Supplementary material for: Management of in-Amphora “Trebbiano Toscano” Wine Production: Selection of Indigenous Saccharomyces cerevisiae Strains and Influence on the Phenolic and Sensory Profile
Source: Foods. 2023 Jun 14;12(12):2372. doi: 10.3390/foods12122372 (PMC10296959; doi:10.3390/foods12122372)
Supplement: Supplementary file 1 [file foods-12-02372-s001.zip › Supplementary Figure caption.pdf]

Figure S1. Workflow of the research.

Figure S2. Heatmap based on indigenous *S. cerevisiae* strains features: killer character (A), capability to produce hydrogen sulphide (B), -glucosidase activity (C), protease activity (D), average isolation frequencies found in spontaneous (E) or inoculated fermentations (F) realized in amphora. The values of each activity were standardized to range from 0 to 100. Colours correspond to standardized values from low (white) to high (black).

Figure S3. Fermentation performances in 20L amphorae of the selected indigenous *S. cerevisiae* strains (AI, AVIII, AXIX) and the commercial strain Anchor VIN13 monitored by quantifying the production of ethanol and the degradation of total sugars during the time.

Figure S4. Principal Component Analysis carried out on the sensory analysis of the wines obtained with different *S. cerevisiae* strains (AI, AVIII, AXIX, VIN13) in 20L amphorae. (a): similarity map determined by Principal Component (Factor) 1 and 2; (b): projection of the variables on the factor plane. Variables: OF: olfactory frankness; OI: olfactory intensity; OQ: olfactory quality; GF: gustatory frankness; GI: gustatory intensity; GQ: gustatory quality; GP: gustatory persistence; G: general impression
