# Supplementary material for: Management of in-Amphora “Trebbiano Toscano” Wine Production: Selection of Indigenous Saccharomyces cerevisiae Strains and Influence on the Phenolic and Sensory Profile
Source: Foods. 2023 Jun 14;12(12):2372. doi: 10.3390/foods12122372 (PMC10296959; doi:10.3390/foods12122372)
Supplement: Supplementary file 1 [file foods-12-02372-s001.zip › Table S2.pdf]

Table S2. Phenolic composition of the wines aged in amphorae was monitored for 12 months. Different letters indicate significant differences among samples collected at a different ageing time from each amphora (ANOVA, Tukey test at p<0.01).

	A1			A3			A5		
months	4	6	12	4	6	12	4	6	12
**Hydroxybenzoic acids and der. (mg/L)**									
gallic acid	8,9	9,4	10,1	10,3	10,4	10,1	10,1	10,1	9,6
protocatecuic acid	0,5	0,9	0,5	0,8	0,3	1,9	0,8	1,1	1,7
vanillic acid	1,3	1,9	1,1	1,7	1,9	1,5	2,2	2,4	2,6
syringic acid	0,4	0,5	0,2	0,5	0,5	0,2	0,6	0,6	0,4
methylgallate	3,4	2,8	1,7	2,6	1,1	1,8	2,5	2,7	1,8
ethylgallate	0,1	0,5	2,1	0,5	0,5	2,0	0,3	0,4	2,0
**Total hydroxybenzoic acids**	**14,6±0.3 a**	**16,0±0.4 a**	**15,8±0.2a**	**16,5±0.2 a**	**14,6±0.3 a**	**17,5±1.4 a**	**16,6±0.3 a**	**17,2±0.3 a**	**18,2±0.3 a**
**Hydroxycinnamic acids and der. (mg/L)**									
caffeic acid	1,7	0,6	0,6	0,7	0,6	0,6	0,8	0,8	0,7
trans-p-coumaric acid	0,5	<0,1	0,7	0,6	0,5	0,7	<0,1	<0,1	0,6
ferulic acid	<0,1	<0,1	<0,1	<0,1	<0,1	0,3	<0,1	<0,1	<0,1
fertaric acid	<0,1	<0,1	<0,1	<0,1	<0,1	<0,1	<0,1	<0,1	<0,1
trans-caftaric acid	2,5	2,4	2,2	2,2	2,2	2,1	2,5	2,9	3,0
cis-p-cutaric acid	1,7	1,6	0,1	1,5	1,3	0,3	2,1	1,7	0,2
trans-p-cutaric acid	0,7	0,4	0,7	0,5	0,2	0,1	0,5	0,5	0,1
**Total hydroxycinnamic acids**	**7,1±0.1b**	**5,0±0.1a**	**4,3±0.1a**	**5,5±0.1b**	**4,8±0.1b**	**4,0±0.0a**	**5,9±0.1b**	**5,9±0.0b**	**4,6±0.0a**
**Stilbenes (mg/L)**									
trans-piceid	0,1	0,2	<0,1	0,1	0,2	<0,1	0,1	0,2	<0,1
cis-piceid	0,1	<0,1	<0,1	0,1	<0,1	<0,1	0,1	0,1	<0,1
trans-resveratrol	0,1	0,1	<0,1	<0,1	0,1	<0,1	<0,1	0,1	<0,1
cis-resveratrol	<0,1	<0,1	<0,1	<0,1	<0,1	<0,1	<0,1	<0,1	<0,1
**Total stilbenes**	**0,3±0.0b**	**0,3±0.0b**	**<0,1a**	**0,2±0.0b**	**0,3±0.0c**	**<0,1a**	**0,2±0.0b**	**0,4±0.0c**	**<0,1a**
**Flavan-3-ols (mg/L)**									
catechin	4,7	5,0	6,2	6,5	5,2	6,3	5,7	5,5	6,0
epicatechin	2,9	3,1	3,1	1,0	3,3	3,0	2,6	2,8	3,3
epicatechin-3-gallate	1,6	1,2	0,2	1,6	1,3	0,2	1,7	1,2	0,3
**Total flavan-3-ols**	**9,2±0.2a**	**9,3±0.0a**	**9,5±0.1a**	**9,1±0.1a**	**9,9±0.0b**	**9,5±0.0a b**	**10,0±0.0 a**	**9,4±0.2a**	**9,6±0.0a**
**Flavonols (mg/L)**									
myricetin-3-galactoside	<0,1	<0,1	<0,1	<0,1	<0,1	<0,1	<0,1	<0,1	<0,1
myricetin-3-glucoside	<0,1	<0,1	<0,1	<0,1	<0,1	<0,1	<0,1	<0,1	<0,1
quercetin-3-galactoside	<0,1	<0,1	<0,1	<0,1	<0,1	0,2	<0,1	<0,1	<0,1
quercetin-3-glucuronide	2,5	2,5	2,3	2,6	2,4	2,3	3,2	2,8	2,8
quercetin-3-glucoside	0,2	0,2	<0,1	0,2	0,2	<0,1	0,3	0,2	<0,1
kaempferol-3-glucoside	<0,1	<0,1	<0,1	<0,1	<0,1	<0,1	<0,1	<0,1	<0,1
myricetin	<0,1	<0,1	<0,1	<0,1	<0,1	<0,1	<0,1	<0,1	<0,1
quercetin	1,1	1,0	1,0	0,9	0,7	0,4	1,0	0,8	0,8
kaempferol	0,4	0,4	0,6	0,4	0,4	0,7	0,6	0,6	0,5
**Total flavonols**	**4,3±0.0b**	**4,1±0.1a**	**3,9±0.0a**	**4,2±0.1b**	**3,7±0.1a**	**3,6±0.0a**	**5,1±0.0b**	**4,4±0.0a**	**4,1±0.1a**
**Phenolic alcohols (mg/L)**									
tyrosol	23,9	22,3	22,8	23,4	22,2	21,9	24,8	24,4	23,5
tryptophol	<0,1	<0,1	0,6	<0,1	<0,1	0,6	<0,1	<0,1	0,6

hydroxytyrosol	0,9	1,0	1,3	1,0	0,9	1,3	1,2	0,8	1,0
**Total phenolic alcohols**	**24,8±1.1 a**	**23,3±1.5 a**	**24,7±01.5 a**	**24,4±0.6 a**	**23,1±0.1 a**	**23,8±1.1 a**	**26,0±0.0 a**	**25,2±0.0 a**	**25,1±0.8 a**
**Volatile phenols (µg/L)**									
4-vinylphenol	103	99	<0.1	97	103	200	3	<0,1	<0,1
4-vinylguaiacol	51	36	<0,1	108	102	21	11	<0,1	14
4-ethylphenol	17	17	62	1	<0,1	36	69	69	97
4-ethylguaiacol	245	235	270	172	160	225	375	361	369
**Total valatile phenols**	**416±66a**	**387±32a**	**332±24a**	**378±29a**	**365±26a**	**482±47a**	**458±52a**	**430±31a**	**480±48a**
