# Supplementary figures and images for: Management of in-Amphora “Trebbiano Toscano” Wine Production: Selection of Indigenous Saccharomyces cerevisiae Strains and Influence on the Phenolic and Sensory Profile
Source: Foods. 2023 Jun 14;12(12):2372. doi: 10.3390/foods12122372 (PMC10296959; doi:10.3390/foods12122372)

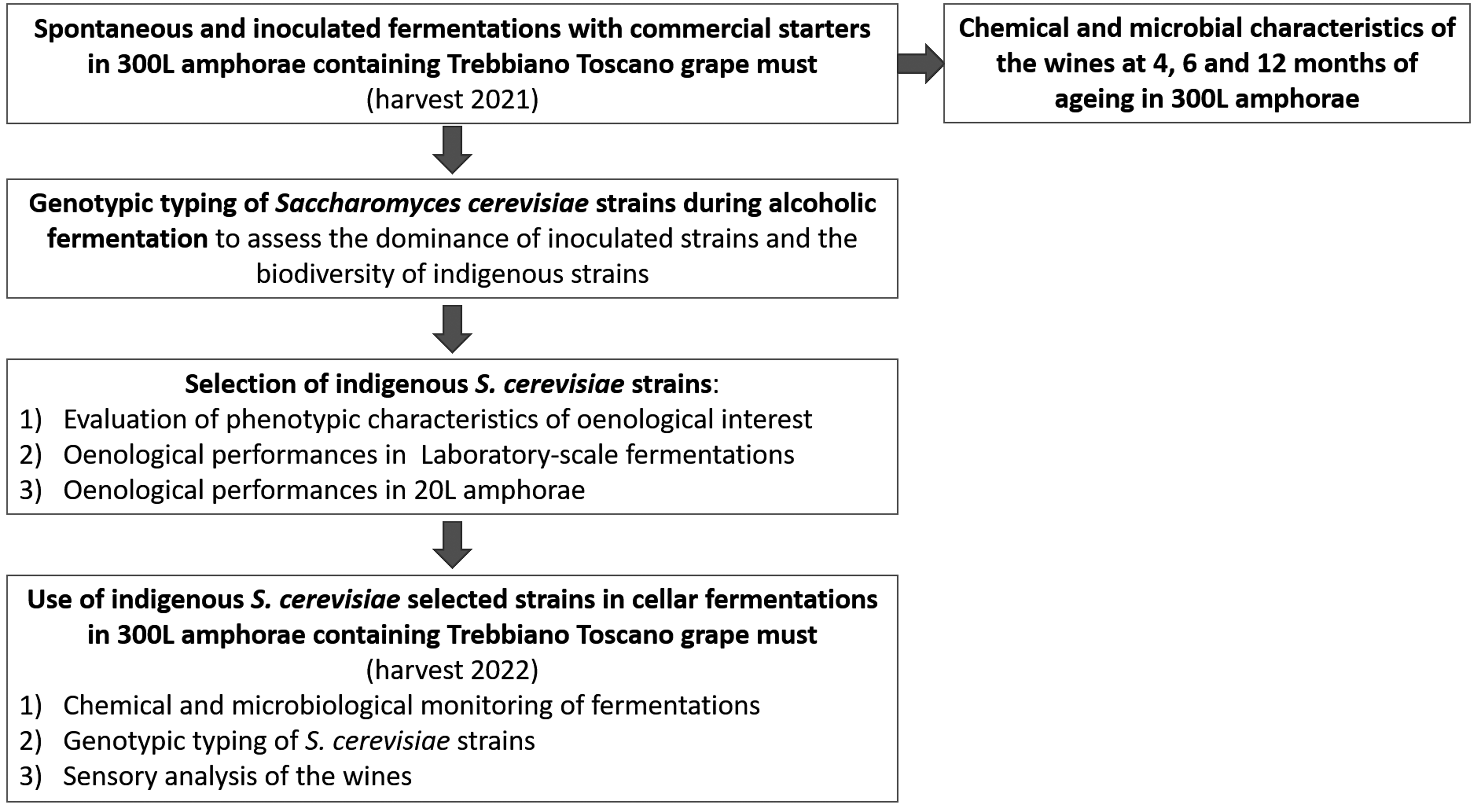

Supplement: Supplementary file 1 [file foods-12-02372-s001.zip › Figure S1.tif]

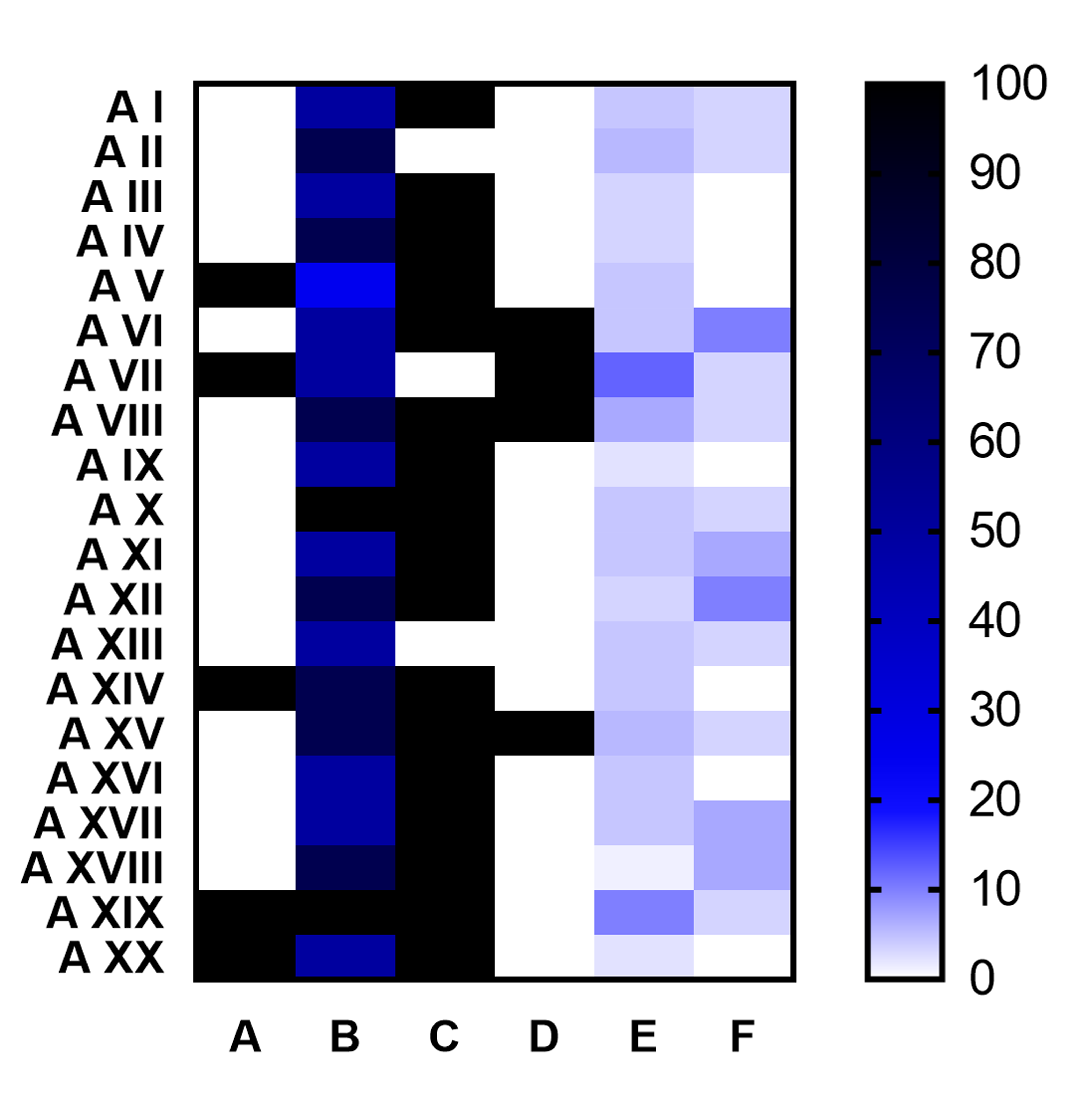

Supplement: Supplementary file 1 [file foods-12-02372-s001.zip › Figure S2.tif]

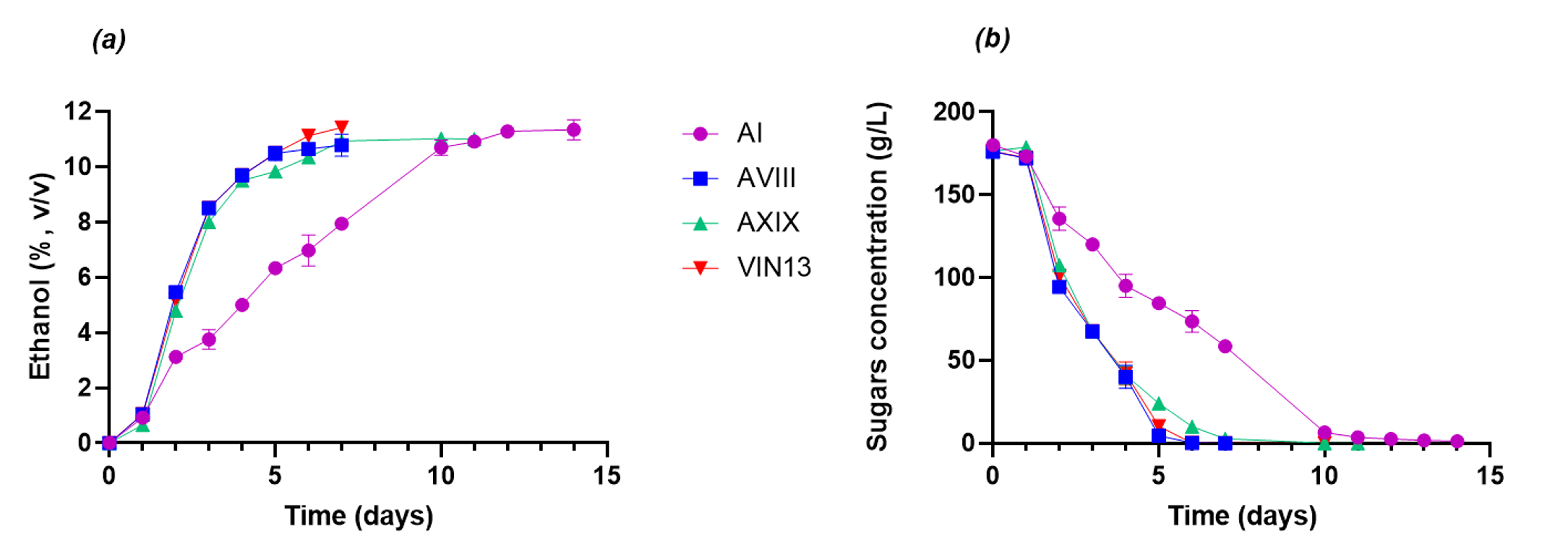

Supplement: Supplementary file 1 [file foods-12-02372-s001.zip › Figure S3.tif]

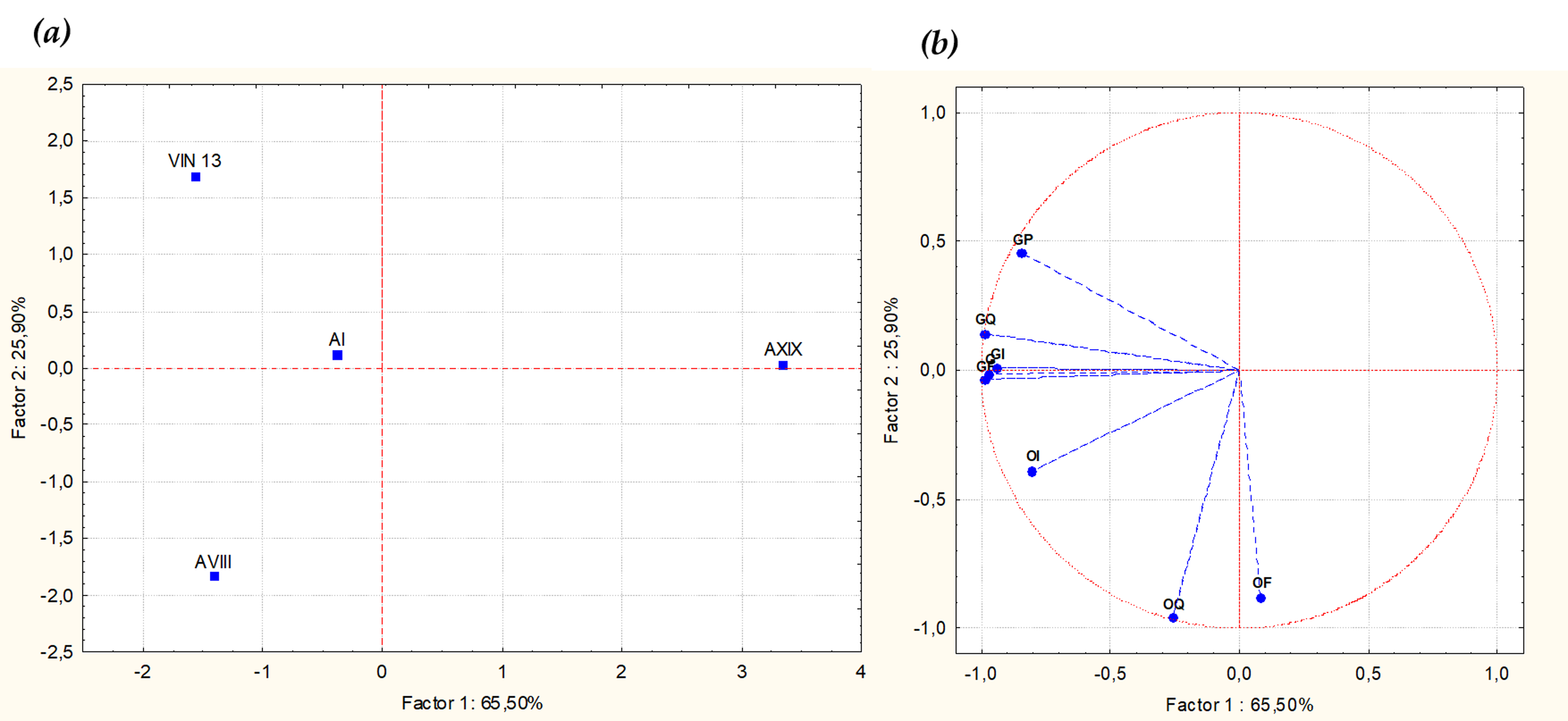

Supplement: Supplementary file 1 [file foods-12-02372-s001.zip › Figure S4.tif]
