# Supplementary material for: Management of in-Amphora “Trebbiano Toscano” Wine Production: Selection of Indigenous Saccharomyces cerevisiae Strains and Influence on the Phenolic and Sensory Profile
Source: Foods. 2023 Jun 14;12(12):2372. doi: 10.3390/foods12122372 (PMC10296959; doi:10.3390/foods12122372)
Supplement: Supplementary file 1 [file Table S1.pdf › Table S1.pdf]

Table S1. Biogenic amines concentrations occurring in the wines during ageing in 300L amphorae. Different letters indicate significant differences among samples collected at a different ageing time from each amphora (ANOVA, Tukey test at p<0.01).

	**A1+2 (Anchor VIN13)**			**A3+4 (Aroma White Enartis)**			**A5+6 (Spontaneous)**		
**Months**	**4**	**6**	**12**	**4**	**6**	**12**	**4**	**6**	**12**
**Agmatine (mg/L)**	2.8±0.1	2.4±0.0	2.6±0.1	2.8±0.0	2.4±0.1	2.4±0.1	2.8±0.0b	1.5±0.1a	2.3±0.1ab
**Ethanolamine (mg/L)**	11.4±0.3b	5.3±0.1a	5.5±0.0a	10.8±0.3b	5.3±0.1a	5.1±0.0a	6.2±0.1b	4.9±0.1a	4.5±0.1a
**Phenylethylamine (mg/L)**	<0.1	<0.1	<0.1	<0.1	<0.1	<0.1	<0.1	<0.1	<0.1
**Putrescine (mg/L)**	1.5±0.1	1.2±0.0	1.6±0.1	1.4±0.3	1.0±0.0	1.6±0.1	0.7±0.0	0.2±0.1	0.6±0.0
**Cadaverine (mg/L)**	<0.1	<0.1	<0.1	<0.1	<0.1	<0.1	<0.1	<0.1	0.1±0.1
**Histamine (mg/L)**	0.5±0.0	0.9±0.1	0.5±0.0	0.5±0.0a	0.9±0.1b	0.5±0.0a	0.4±0.1	0.8±0.0	0.2±0.1
**Tyramine (mg/L)**	<0.1	<0.1	0.1	<0.1	<0.1	0.1±0.1	<0.1	<0.1	0.1±0.0
**Spermidine (mg/L)**	<0.1	<0.1	<0.1	<0.1	<0.1	<0.1	<0.1	<0.1	<0.1
**Spermine (mg/L)**	<0.1	<0.1	<0.1	<0.1	<0.1	<0.1	<0.1	<0.1	<0.1
